# (±)-*syn*-Isopropyl 4-(1,1,1,3,3,3-hexa­fluoro­propan-2-yl­oxy)-1-hydr­oxy-3-methyl-2-(prop-1-yn­yl)cyclo­pent-2-ene­carboxyl­ate

**DOI:** 10.1107/S1600536809023162

**Published:** 2009-06-20

**Authors:** Annika Gille, Markus Schürmann, Hans Preut, Martin Hiersemann

**Affiliations:** a Fakultät Chemie, Technische Universität Dortmund, Otto-Hahn-Strasse 6, 44221 Dortmund, Germany

## Abstract

The title compound, C_16_H_18_F_6_O_4_, was obtained through an unprecedented one-pot reaction sequence involving a Gosteli–Claisen rearrangement and a cyclo­isomerization. The constitution and relative configuration were determined by single-crystal X-ray diffraction analysis. In the crystal, mol­ecules are connected *via* O—H ⋯ O hydrogen bonds.

## Related literature

For the preparation, see:  Neises & Steglich (1978 ▶); Hiersemann (2000 ▶). For details of the Gosteli–Claisen rearrangement, see: Gosteli (1972 ▶); Landor & Black (1965 ▶).
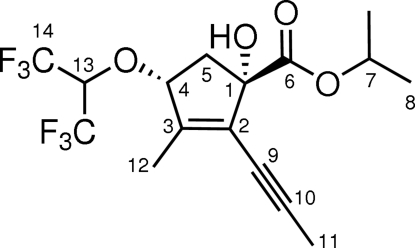


## Experimental

### 

#### Crystal data

C_16_H_18_F_6_O_4_
*M* *_r_* = 388.30
Triclinic, 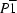

*a* = 6.0166 (4) Å
*b* = 11.9075 (6) Å
*c* = 13.2798 (8) Å
α = 104.600 (5)°
β = 91.775 (5)°
γ = 96.955 (5)°
*V* = 912.03 (10) Å^3^
*Z* = 2
Mo *K*α radiation
μ = 0.14 mm^−1^
*T* = 173 K
0.42 × 0.20 × 0.18 mm

#### Data collection

Oxford Diffraction Xcalibur S CCD diffractometer
Absorption correction: multi-scan (*CrysAlis RED*; Oxford Diffraction, 2008 ▶) *T*_min_ = 0.944, *T*_max_ = 0.975
7349 measured reflections
3175 independent reflections
2089 reflections with *I* > 2σ(*I*)
*R*_int_ = 0.026

#### Refinement

*R*[*F* ^2^ > 2σ(*F* ^2^)] = 0.042
*wR*(*F* ^2^) = 0.106
*S* = 0.96
3175 reflections
240 parameters
H-atom parameters constrained
Δρ_max_ = 0.27 e Å^−3^
Δρ_min_ = −0.29 e Å^−3^

### 

Data collection: *CrysAlis CCD* (Oxford Diffraction, 2008 ▶); cell refinement: *CrysAlis RED* (Oxford Diffraction, 2008 ▶); data reduction: *CrysAlis RED*; program(s) used to solve structure: *SHELXS97* (Sheldrick, 2008 ▶); program(s) used to refine structure: *SHELXL97* (Sheldrick, 2008 ▶); molecular graphics: *SHELXTL-Plus* (Sheldrick, 2008 ▶); software used to prepare material for publication: *SHELXL97* and *PLATON* (Spek, 2009 ▶).

## Supplementary Material

Crystal structure: contains datablocks I, global. DOI: 10.1107/S1600536809023162/hg2521sup1.cif

Structure factors: contains datablocks I. DOI: 10.1107/S1600536809023162/hg2521Isup2.hkl

Additional supplementary materials:  crystallographic information; 3D view; checkCIF report

## Figures and Tables

**Table 1:** Hydrogen-bond geometry (Å, °)

*D*—H⋯*A*	*D*—H	H⋯*A*	*D*⋯*A*	*D*—H⋯*A*
O1—H1⋯O3^i^	0.84	2.10	2.8431 (17)	148

Symmetry code: (i) 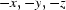.

**Fig. 1.**
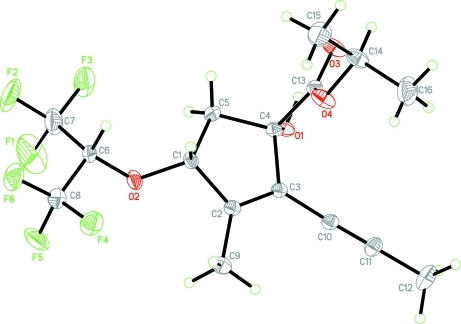
The molecular structure of (I) with displacement ellipsoids shown at the 30% probability level.

## supplementary crystallographic information

### Comment

For the preparation of the title compound (±)-(I) a propargyl vinyl ether (II) was synthesized from 2-butin-1-ol using an etherification with bromoacetic acid, an esterification under Steglich's conditions (Neises & Steglich, 1978) and an aldol condensation (Hiersemann, 2000). An unprecedented one-pot reaction sequence involving an uncatalyzed Gosteli-Claisen rearrangement (Gosteli, 1972) of the propargyl vinyl ether (II) (Landor & Black, 1965) and a cycloisomerization under incorporation of a solvent molecule provided (I) as a colourless solid. Fig. 1 depicts the constitution and relative configuration of the isolated diastereomer (I).

### Experimental

The title compound (I) was synthesized according to the following procedure. A solution of (II) (0.044 g, 0.2 mmol) in dry hexafluoroisopropanol (0.6 ml) was stirred at 333 K for 23 h. The solvent was removed under reduced pressure. Flash chromatography (isohexane/ethyl acetate 100/1 to 50/1 to 20/1) afforded (I) as a single diastereomer (0.026 g, 0.07 mmol, 34%) and 37% of the starting material (II). Single crystals of (I) were obtained by vapor diffusion recrystallization technique from isohexane and ethyl acetate to yield colorless cuboids: mp 385 K; *R*_f_ 0.32 (cyclohexane/ethyl acetate 5/1); ^1^H NMR (CDCl_3_, 400 MHz, δ): 1.23 (d, *J* = 6.3 Hz, 3H, 8-H), 1.26 (d, *J* = 6.3 Hz, 3H, 8-H), 1.91 (s, 3H, 12-H), 2.01 (s, 3H, 11-H), 2.03 (dd, *J* = 13.6, 5.9 Hz, 1H, 5-H), 2.86 (dd, *J* = 13.6, 7.0 Hz, 1H, 5-H), 3.76 (br.s, 1H, –OH), 4.23 (sep, *J* = 5.9 Hz, 1H, 13-H), 4.67 (t, *J* = 5.9 Hz, 1H, 4-H), 5.06 (sep, *J* = 6.3 Hz, 1H, 7-H); ^19^F NMR (CDCl_3_, 282 MHz, δ): (-77.0)-(-76.7) (m, 6 F); ^13^C NMR (CDCl_3_, 101 MHz, δ): 4.8 (11-CH_3_), 13.3 (12-CH_3_), 21.7 (8-CH_3_), 21.8 (8-CH_3_), 42.7 (5-CH_2_), 70.6 (7-CH), 71.6 (9-C), 76.0 (sep, *J* = 32.6 Hz, 13-CH), 83.1 (1-C), 88.9 (4-CH), 94.5 (10-C), 121.2 (q, *J* = 283.0 Hz, 14-C), 121.8 (q, *J* = 283.0 Hz, 14-C), 126.5 (2-C), 146.9 (3-C), 173.6 (6-C); IR (cm^-1^): 3485(br,s) (ν O—H, OH in H-bridges), 2990(m) 2940(m) 2920(m) (ν_as,s_ C—H, CH_2_, CH_3_, CH), 2230(w) (ν C≡C), 1715(s) (ν C=O, ester), 1635(m) (ν C=C), 1380(m) (δ_s_ C—H, CH_3_), 1295(s) (ν C—O, ester), 1240(s) 1230(s) 1220(s) (ν C—F), 1185(s) 1120(s) 1105(s) (ν C—O, ether); Anal. Calcd. for C_16_H_18_F_6_O_4_: C,49.5; H, 4.7; Found: C, 49.1; H, 4.7; M = 388.30 g/mol.

### Crystal data

**Table:**

C_16_H_18_F_6_O_4_	*Z* = 2
*M**_r_* = 388.30	*F*(000) = 400
Triclinic, *P*1	*D*_x_ = 1.414 Mg m^−^^3^
Hall symbol: -P 1	Mo *K*α radiation, λ = 0.71073 Å
*a* = 6.0166 (4) Å	Cell parameters from 3408 reflections
*b* = 11.9075 (6) Å	θ = 2.7–25.0°
*c* = 13.2798 (8) Å	µ = 0.14 mm^−^^1^
α = 104.600 (5)°	*T* = 173 K
β = 91.775 (5)°	Block, colourless
γ = 96.955 (5)°	0.42 × 0.20 × 0.18 mm
*V* = 912.03 (10) Å^3^	

### Data collection

**Table:**

Oxford Diffraction Xcalibur S CCD diffractometer	3175 independent reflections
Radiation source: Enhance (Mo) X-ray Source	2089 reflections with *I* > 2σ(*I*)
graphite	*R*_int_ = 0.026
Detector resolution: 16.0560 pixels mm^-1^	θ_max_ = 25.0°, θ_min_ = 2.7°
ω scans	*h* = −7→7
Absorption correction: multi-scan (*CrysAlis RED*; Oxford Diffraction, 2008)	*k* = −13→14
*T*_min_ = 0.944, *T*_max_ = 0.975	*l* = −15→11
7349 measured reflections	

### Refinement

**Table:**

Refinement on *F*^2^	Primary atom site location: structure-invariant direct methods
Least-squares matrix: full	Secondary atom site location: difference Fourier map
*R*[*F*^2^ > 2σ(*F*^2^)] = 0.042	Hydrogen site location: inferred from neighbouring sites
*wR*(*F*^2^) = 0.106	H-atom parameters constrained
*S* = 0.96	*w* = 1/[σ^2^(*F*_o_^2^) + (0.0599*P*)^2^] where *P* = (*F*_o_^2^ + 2*F*_c_^2^)/3
3175 reflections	(Δ/σ)_max_ < 0.001
240 parameters	Δρ_max_ = 0.27 e Å^−^^3^
0 restraints	Δρ_min_ = −0.29 e Å^−^^3^

### Special details

**Table:**

Experimental. CrysAlis RED (Oxford Diffraction 2008) Empirical absorption correction using spherical harmonics, implemented in SCALE3 ABSPACK scaling algorithm.
Geometry. All e.s.d.'s (except the e.s.d. in the dihedral angle between two l.s. planes) are estimated using the full covariance matrix. The cell e.s.d.'s are taken into account individually in the estimation of e.s.d.'s in distances, angles and torsion angles; correlations between e.s.d.'s in cell parameters are only used when they are defined by crystal symmetry. An approximate (isotropic) treatment of cell e.s.d.'s is used for estimating e.s.d.'s involving l.s. planes.
Refinement. Refinement of *F*^2^ against ALL reflections. The weighted *R*-factor *wR* and goodness of fit *S* are based on *F*^2^, conventional *R*-factors *R* are based on *F*, with *F* set to zero for negative *F*^2^. The threshold expression of *F*^2^ > σ(*F*^2^) is used only for calculating *R*-factors(gt) *etc*. and is not relevant to the choice of reflections for refinement. *R*-factors based on *F*^2^ are statistically about twice as large as those based on *F*, and *R*- factors based on ALL data will be even larger.

### Fractional atomic coordinates and isotropic or equivalent isotropic displacement parameters (Å^2^)

**Table:**

	*x*	*y*	*z*	*U*_iso_*/*U*_eq_	
F1	0.8004 (4)	0.56391 (17)	0.16252 (16)	0.1130 (8)	
F2	1.0918 (3)	0.49063 (18)	0.10772 (12)	0.1036 (7)	
F3	0.7665 (3)	0.39187 (15)	0.06173 (11)	0.0842 (6)	
F4	1.0556 (2)	0.43622 (12)	0.40071 (10)	0.0537 (4)	
F5	0.9672 (2)	0.59099 (11)	0.36511 (11)	0.0631 (5)	
F6	1.2566 (2)	0.51846 (13)	0.30212 (11)	0.0608 (4)	
O1	0.0648 (2)	0.15814 (11)	0.13523 (10)	0.0286 (4)	
H1	0.0264	0.1079	0.0786	0.043*	
O2	0.6874 (2)	0.38842 (11)	0.26452 (11)	0.0310 (4)	
O3	0.1934 (2)	−0.05252 (12)	0.05543 (11)	0.0406 (4)	
O4	0.4173 (2)	−0.03491 (12)	0.19701 (11)	0.0391 (4)	
C1	0.6115 (3)	0.26537 (16)	0.25431 (15)	0.0243 (5)	
H1A	0.7429	0.2231	0.2609	0.029*	
C2	0.4539 (3)	0.25339 (16)	0.33674 (15)	0.0243 (5)	
C3	0.2714 (3)	0.17686 (16)	0.29615 (15)	0.0231 (4)	
C4	0.2677 (3)	0.13463 (16)	0.17825 (14)	0.0227 (4)	
C5	0.4762 (3)	0.20733 (17)	0.15116 (15)	0.0293 (5)	
H5A	0.5661	0.1562	0.1028	0.035*	
H5B	0.4311	0.2673	0.1177	0.035*	
C6	0.9030 (3)	0.41341 (17)	0.23280 (16)	0.0299 (5)	
H6	0.9684	0.3384	0.2098	0.036*	
C7	0.8883 (5)	0.4666 (2)	0.1413 (2)	0.0605 (8)	
C8	1.0461 (4)	0.4918 (2)	0.32484 (18)	0.0397 (6)	
C9	0.5107 (3)	0.31553 (18)	0.44862 (15)	0.0367 (6)	
H9A	0.3943	0.2902	0.4912	0.055*	
H9B	0.5193	0.4002	0.4574	0.055*	
H9C	0.6557	0.2970	0.4708	0.055*	
C10	0.0936 (3)	0.13487 (17)	0.35228 (15)	0.0256 (5)	
C11	−0.0581 (3)	0.09773 (18)	0.39548 (16)	0.0318 (5)	
C12	−0.2433 (4)	0.0527 (2)	0.44912 (19)	0.0486 (6)	
H12A	−0.2256	−0.0270	0.4525	0.073*	
H12B	−0.3858	0.0518	0.4109	0.073*	
H12C	−0.2428	0.1032	0.5200	0.073*	
C13	0.2847 (3)	0.00474 (17)	0.13700 (15)	0.0237 (5)	
C14	0.4642 (4)	−0.15683 (18)	0.16071 (17)	0.0392 (6)	
H14	0.3778	−0.1949	0.0926	0.047*	
C15	0.7079 (4)	−0.1520 (2)	0.1452 (2)	0.0602 (8)	
H15A	0.7475	−0.1045	0.0963	0.090*	
H15B	0.7441	−0.2315	0.1168	0.090*	
H15C	0.7929	−0.1168	0.2121	0.090*	
C16	0.3840 (5)	−0.2186 (2)	0.2411 (2)	0.0710 (9)	
H16A	0.4691	−0.1819	0.3078	0.107*	
H16B	0.4064	−0.3011	0.2184	0.107*	
H16C	0.2241	−0.2131	0.2495	0.107*	

### Atomic displacement parameters (Å^2^)

**Table:**

	*U*^11^	*U*^22^	*U*^33^	*U*^12^	*U*^13^	*U*^23^
F1	0.179 (2)	0.0561 (12)	0.1164 (16)	0.0058 (12)	−0.0384 (15)	0.0547 (11)
F2	0.1085 (14)	0.1364 (17)	0.0573 (11)	−0.0682 (13)	0.0018 (10)	0.0494 (11)
F3	0.1084 (12)	0.0924 (13)	0.0418 (9)	−0.0499 (10)	−0.0191 (9)	0.0316 (8)
F4	0.0516 (8)	0.0668 (10)	0.0425 (8)	−0.0019 (7)	−0.0024 (6)	0.0191 (7)
F5	0.0721 (9)	0.0333 (8)	0.0679 (10)	0.0027 (7)	0.0029 (8)	−0.0145 (7)
F6	0.0357 (8)	0.0757 (10)	0.0675 (10)	−0.0151 (7)	0.0060 (7)	0.0220 (8)
O1	0.0311 (7)	0.0296 (8)	0.0227 (8)	0.0091 (6)	−0.0052 (6)	0.0008 (6)
O2	0.0303 (8)	0.0201 (7)	0.0417 (9)	0.0021 (6)	0.0045 (6)	0.0069 (6)
O3	0.0513 (9)	0.0306 (8)	0.0333 (9)	0.0110 (7)	−0.0156 (8)	−0.0043 (7)
O4	0.0503 (9)	0.0288 (8)	0.0346 (9)	0.0142 (7)	−0.0144 (7)	−0.0006 (7)
C1	0.0238 (10)	0.0183 (10)	0.0301 (11)	0.0032 (8)	0.0018 (9)	0.0050 (8)
C2	0.0275 (10)	0.0239 (10)	0.0219 (11)	0.0061 (8)	0.0003 (9)	0.0058 (8)
C3	0.0251 (10)	0.0231 (10)	0.0214 (10)	0.0055 (8)	0.0020 (8)	0.0051 (8)
C4	0.0215 (10)	0.0265 (10)	0.0198 (10)	0.0046 (8)	0.0000 (8)	0.0050 (8)
C5	0.0327 (11)	0.0312 (11)	0.0228 (11)	−0.0001 (9)	0.0052 (9)	0.0065 (9)
C6	0.0355 (11)	0.0199 (10)	0.0337 (12)	−0.0011 (8)	0.0092 (10)	0.0073 (9)
C7	0.0795 (19)	0.0498 (18)	0.0488 (17)	−0.0232 (15)	−0.0063 (15)	0.0228 (14)
C8	0.0365 (13)	0.0399 (14)	0.0404 (14)	−0.0015 (10)	0.0045 (11)	0.0089 (11)
C9	0.0404 (12)	0.0375 (13)	0.0276 (12)	0.0012 (10)	−0.0024 (10)	0.0022 (10)
C10	0.0291 (11)	0.0266 (11)	0.0219 (11)	0.0053 (9)	−0.0001 (9)	0.0070 (9)
C11	0.0309 (12)	0.0387 (13)	0.0294 (12)	0.0042 (10)	0.0011 (10)	0.0157 (10)
C12	0.0402 (13)	0.0657 (17)	0.0502 (16)	0.0040 (12)	0.0107 (12)	0.0342 (13)
C13	0.0183 (9)	0.0294 (11)	0.0221 (11)	0.0012 (8)	0.0025 (9)	0.0052 (9)
C14	0.0536 (14)	0.0256 (12)	0.0372 (13)	0.0147 (10)	−0.0072 (11)	0.0026 (10)
C15	0.0656 (17)	0.0441 (15)	0.078 (2)	0.0211 (13)	0.0294 (15)	0.0188 (14)
C16	0.079 (2)	0.0405 (15)	0.100 (2)	0.0080 (14)	0.0324 (18)	0.0276 (16)

### Geometric parameters (Å, °)

**Table:**

F1—C7	1.302 (3)	C5—H5A	0.9900
F2—C7	1.339 (3)	C5—H5B	0.9900
F3—C7	1.329 (3)	C6—C8	1.507 (3)
F4—C8	1.341 (3)	C6—C7	1.510 (3)
F5—C8	1.319 (2)	C6—H6	1.0000
F6—C8	1.330 (2)	C9—H9A	0.9800
O1—C4	1.419 (2)	C9—H9B	0.9800
O1—H1	0.8400	C9—H9C	0.9800
O2—C6	1.397 (2)	C10—C11	1.192 (3)
O2—C1	1.452 (2)	C11—C12	1.461 (3)
O3—C13	1.200 (2)	C12—H12A	0.9800
O4—C13	1.318 (2)	C12—H12B	0.9800
O4—C14	1.474 (2)	C12—H12C	0.9800
C1—C2	1.492 (3)	C14—C15	1.483 (3)
C1—C5	1.528 (3)	C14—C16	1.502 (4)
C1—H1A	1.0000	C14—H14	1.0000
C2—C3	1.342 (2)	C15—H15A	0.9800
C2—C9	1.492 (3)	C15—H15B	0.9800
C3—C10	1.434 (3)	C15—H15C	0.9800
C3—C4	1.517 (3)	C16—H16A	0.9800
C4—C13	1.522 (3)	C16—H16B	0.9800
C4—C5	1.541 (3)	C16—H16C	0.9800
			
C4—O1—H1	109.5	F5—C8—F4	106.86 (19)
C6—O2—C1	115.42 (14)	F6—C8—F4	106.70 (18)
C13—O4—C14	118.33 (14)	F5—C8—C6	113.48 (18)
O2—C1—C2	109.65 (14)	F6—C8—C6	112.73 (19)
O2—C1—C5	112.11 (17)	F4—C8—C6	109.13 (18)
C2—C1—C5	105.10 (14)	C2—C9—H9A	109.5
O2—C1—H1A	110.0	C2—C9—H9B	109.5
C2—C1—H1A	110.0	H9A—C9—H9B	109.5
C5—C1—H1A	110.0	C2—C9—H9C	109.5
C3—C2—C9	127.42 (19)	H9A—C9—H9C	109.5
C3—C2—C1	110.79 (16)	H9B—C9—H9C	109.5
C9—C2—C1	121.69 (16)	C11—C10—C3	177.6 (2)
C2—C3—C10	126.87 (18)	C10—C11—C12	179.6 (2)
C2—C3—C4	112.41 (17)	C11—C12—H12A	109.5
C10—C3—C4	120.72 (15)	C11—C12—H12B	109.5
O1—C4—C3	108.58 (14)	H12A—C12—H12B	109.5
O1—C4—C13	108.35 (13)	C11—C12—H12C	109.5
C3—C4—C13	114.56 (17)	H12A—C12—H12C	109.5
O1—C4—C5	112.55 (16)	H12B—C12—H12C	109.5
C3—C4—C5	103.23 (14)	O3—C13—O4	124.56 (18)
C13—C4—C5	109.61 (15)	O3—C13—C4	122.24 (17)
C1—C5—C4	106.17 (16)	O4—C13—C4	113.11 (15)
C1—C5—H5A	110.5	O4—C14—C15	107.02 (18)
C4—C5—H5A	110.5	O4—C14—C16	106.87 (18)
C1—C5—H5B	110.5	C15—C14—C16	115.0 (2)
C4—C5—H5B	110.5	O4—C14—H14	109.3
H5A—C5—H5B	108.7	C15—C14—H14	109.3
O2—C6—C8	108.60 (17)	C16—C14—H14	109.3
O2—C6—C7	109.24 (18)	C14—C15—H15A	109.5
C8—C6—C7	113.26 (19)	C14—C15—H15B	109.5
O2—C6—H6	108.5	H15A—C15—H15B	109.5
C8—C6—H6	108.5	C14—C15—H15C	109.5
C7—C6—H6	108.5	H15A—C15—H15C	109.5
F1—C7—F3	108.0 (2)	H15B—C15—H15C	109.5
F1—C7—F2	106.8 (2)	C14—C16—H16A	109.5
F3—C7—F2	106.9 (2)	C14—C16—H16B	109.5
F1—C7—C6	113.5 (2)	H16A—C16—H16B	109.5
F3—C7—C6	110.3 (2)	C14—C16—H16C	109.5
F2—C7—C6	111.1 (2)	H16A—C16—H16C	109.5
F5—C8—F6	107.57 (18)	H16B—C16—H16C	109.5
			
C6—O2—C1—C2	149.56 (16)	O2—C6—C7—F1	60.4 (2)
C6—O2—C1—C5	−94.11 (18)	C8—C6—C7—F1	−60.8 (3)
O2—C1—C2—C3	133.92 (16)	O2—C6—C7—F3	−60.9 (3)
C5—C1—C2—C3	13.2 (2)	C8—C6—C7—F3	177.9 (2)
O2—C1—C2—C9	−49.5 (2)	O2—C6—C7—F2	−179.27 (18)
C5—C1—C2—C9	−170.23 (17)	C8—C6—C7—F2	59.6 (3)
C9—C2—C3—C10	−2.3 (3)	O2—C6—C8—F5	−58.6 (2)
C1—C2—C3—C10	173.99 (18)	C7—C6—C8—F5	63.0 (3)
C9—C2—C3—C4	178.06 (18)	O2—C6—C8—F6	178.82 (17)
C1—C2—C3—C4	−5.6 (2)	C7—C6—C8—F6	−59.6 (3)
C2—C3—C4—O1	−123.92 (17)	O2—C6—C8—F4	60.5 (2)
C10—C3—C4—O1	56.4 (2)	C7—C6—C8—F4	−178.0 (2)
C2—C3—C4—C13	114.83 (18)	C14—O4—C13—O3	2.1 (3)
C10—C3—C4—C13	−64.8 (2)	C14—O4—C13—C4	−174.52 (16)
C2—C3—C4—C5	−4.3 (2)	O1—C4—C13—O3	25.9 (2)
C10—C3—C4—C5	176.06 (16)	C3—C4—C13—O3	147.25 (18)
O2—C1—C5—C4	−134.32 (16)	C5—C4—C13—O3	−97.3 (2)
C2—C1—C5—C4	−15.27 (19)	O1—C4—C13—O4	−157.37 (16)
O1—C4—C5—C1	128.89 (16)	C3—C4—C13—O4	−36.0 (2)
C3—C4—C5—C1	12.02 (18)	C5—C4—C13—O4	79.5 (2)
C13—C4—C5—C1	−110.46 (17)	C13—O4—C14—C15	115.9 (2)
C1—O2—C6—C8	−119.38 (18)	C13—O4—C14—C16	−120.4 (2)
C1—O2—C6—C7	116.65 (19)		

### Hydrogen-bond geometry (Å, °)

**Table:**

*D*—H···*A*	*D*—H	H···*A*	*D*···*A*	*D*—H···*A*
O1—H1···O3^i^	0.84	2.10	2.8431 (17)	148

Symmetry codes: (i) −*x*, −*y*, −*z*.
